# Protocol of the QUATTRO-II study: a multicenter randomized phase II study comparing CAPOXIRI plus bevacizumab with FOLFOXIRI plus bevacizumab as a first-line treatment in patients with metastatic colorectal cancer

**DOI:** 10.1186/s12885-020-07186-5

**Published:** 2020-07-23

**Authors:** Masaaki Miyo, Takeshi Kato, Takayuki Yoshino, Takeharu Yamanaka, Hideaki Bando, Hironaga Satake, Kentaro Yamazaki, Hiroya Taniguchi, Eiji Oki, Masahito Kotaka, Koji Oba, Yoshinori Miyata, Kei Muro, Yoshito Komatsu, Hideo Baba, Akihito Tsuji

**Affiliations:** 1 Department of Surgery, National Hospital Organization Osaka National Hospital, 2-1-14, Hoenzaka, Chuo-ku, Osaka, 540-0006 Japan; 2 Department of Gastroenterology and Gastrointestinal Oncology, National Cancer Center Hospital East, Kashiwa City, Japan; 3 Department of Biostatistics, Yokohama City University School of Medicine, Yokohama, Japan; 4 Department of Clinical Oncology, Aichi Cancer Center Hospital, Nagoya, Japan; 5 Cancer Treatment Center, Kansai Medical University Hospital, Hirakata City, Japan; 6 Division of Gastrointestinal Oncology, Shizuoka Cancer Center, Shizuoka, Japan; 7 Department of Surgery and Science, Graduate School of Medical Sciences, Kyushu University, Fukuoka, Japan; 8 Gastrointestinal cancer center, Sano Hospital, Kobe, Japan; 9 Interfaculty Initiative in Information Studies, The University of Tokyo, Tokyo, Japan; 10 Saku Central Hospital Advanced Care Center, Saku City, Japan; 11 Department of Cancer Chemotherapy, Hokkaido University Hospital Cancer Center, Sapporo, Japan; 12 Department of Gastroenterological Surgery Graduate School of Medical Sciences Kumamoto University, Kumamoto, Japan; 13 Department of Medical Oncology, Kagawa University Hospital, Kagawa, Japan

**Keywords:** Colorectal cancer, CAPOXIRI, FOLFOXIRI, Bevacizumab, Randomized, First-line treatment, Dose confirmation, Multicenter, Progression-free survival, Safety

## Abstract

**Background:**

First-line treatment with FOLFOXIRI plus bevacizumab (BEV) is highly effective and regarded as one of the standards-of-care for patients with metastatic colorectal cancer (mCRC), despite the high incidence of neutropenia and diarrhea as side effects. AXEPT, an Asian phase III study, showed that modified CAPIRI+BEV [capecitabine (CAP: 1600 mg/m^2^), irinotecan (IRI: 200 mg/m^2^), and BEV (7.5 mg/m^2^)] was non-inferior to FOLFIRI+BEV as a second-line therapy for mCRC patients and was associated with a lower incidence of hematologic toxicities. Thus, a reduced dose of the CAP and IRI regimen in combination with oxaliplatin (OX) and BEV (CAPOXIRI+BEV) may be more feasible than FOLFOXIRI+BEV, without compromising efficacy.

**Methods:**

QUATTRO-II is an open-label, multicenter, randomized phase II study. In Step 1, the recommended doses of OX and IRI will be investigated as a safety lead-in. In Step 2, patients will be randomized to the recommended dose of either CAPOXIRI+BEV or FOLFOXIRI+BEV. Induction triplet chemotherapy plus BEV treatments will be administered for up to 4 months followed by fluoropyrimidine plus BEV maintenance. The primary endpoint is progression-free survival (PFS). The similarity in PFS between the two arms will be evaluated by observing whether the point estimate of hazard ratio (HR) for PFS falls between 0.80 and 1.25. Ensuring a 70% probability that the observed HR will be “0.8 < HR < 1.25” under the assumption of the true HR of 1.0, and 100 patients will be evaluated during the 3-year study period. Secondary endpoints include overall survival, overall response rate, safety, and patient reported outcome (PRO) (FACT/GOG-Ntx4).

**Discussion:**

Considering the lower incidence of hematologic toxicities with modified CAPIRI+BEV than with FOLFIRI+BEV, CAPOXIRI+BEV may be a promising treatment option if sufficient efficacy and lower hematologic toxicities are indicated in this study. Additionally, a lower incidence of peripheral sensory neuropathy (PSN) reported following CAPEOX treatment compared to that after FOLFOX in ACHIEVE, an adjuvant phase III trial, suggest that CAPOXIRI+BEV can mitigate OX-induced PSN.

**Trial registration:**

Clinicaltrials.gov NCT04097444. Registered September 20, 2019, https://clinicaltrials.gov/ct2/show/study/NCT04097444/ Japan Registry of Clinical Trials jRCTs041190072. Registered October 9, 2019.

## Background

According to National Comprehensive Cancer Network and Japanese Society for Cancer of the Colon and Rectum Guidelines, dual combinations of cytotoxic drugs, such as FOLFOX (oxaliplatin (OX) + 5-fluorouracil (FU)/levofolinate calcium (*l*-LV)), CAPEOX (OX+capecitabine (CAP)), or FOLFIRI (irinotecan (IRI) + 5-FU/*l*-LV), plus molecular targeted agents, such as anti-VEGF antibody or anti-EGFR antibody (only *RAS* wild-type) are frequently used as the first-line regimens for patients with metastatic colorectal cancer (mCRC) [1–9]. For patients with favorable general conditions who require stronger tumor shrinkage and longer tumor controls, FOLFOXIRI, a triple combination consisting of OX, IRI, and 5-FU/*l*-LV, plus bevacizumab (BEV) is an alternative treatment option [10, 11].

The efficacies of FOLFOXIRI+BEV compared with FOLFIRI+BEV as a first-line treatment for mCRC were investigated and demonstrated in the phase III TRIBE Study, validating the significantly better progression-free survival (PFS) and over survival (OS) (median PFS, 12.3 versus 9.7 months; hazard ratio (HR) 0.77, 95% CI 0.65–0.93; *p* = 0.006) (median OS, 29.8 versus 25.8 months; HR 0.80, 95% CI 0.65–0.98; *p* = 0.03). Post-hoc sub-analysis also indicated that FOLFOXIRI+BEV was remarkably effective for improving OS (HR 0.54) in a *BRAF*-mutated population with a poor prognosis, and thus is the first choice for these cases [11, 12]. However, the higher incidence of grade 3 or 4 neutropenia (50.0%), diarrhea (18.8%), and stomatitis (8.8%) may limit the applications of this regimen [10]. In Japan, the feasibility, safety, and efficacy of FOLFOXIRI+BEV were investigated in a single-arm phase II study (QUATTRO study). Although a PFS of 13.3 months and overall response rate (ORR) of 72.1% were observed [13], higher incidences of grade 3 or 4 neutropenia (72.5%), leucopenia (33.3%), and febrile neutropenia (21.7%) were observed in the Japanese population.

CAPEOX and CAPIRI are alternative tri-weekly treatment options without requiring a central venous access port and infusion pump, making these treatments more convenient and cost-effective than bi-weekly FOLFOX and FOLFIRI. Furthermore, an Asian phase III clinical trial (AXEPT) from Japan, China, and South Korea demonstrated that modified CAPIRI+BEV (CAP 1600 mg/m^2^/day, IRI 200 mg/m^2^, and BEV 7.5 mg/m^2^) was non-inferior as a second-line therapy to FOLFIRI+BEV in terms of OS, with a lower incidence of severe neutropenia (grade 3 or 4 neutropenia, 16.8% versus 42.9%) as a second-line treatment [14].

These results suggest that treatment with a reduced dose of CAP in combination with OX/IRI/BEV (CAPOXIRI+BEV) can manage hematologic toxicities without impairing efficacy compared to FOLFOXIRI+BEV. Although a Japanese phase I study conducted by Sato et al. investigated the recommended doses (RDs) of CAPOXIRI+BEV, dose limiting toxicities (DLTs) were not observed at the originally planned maximum dose (CAP 1700 mg/m^2^/day, IRI 150 mg/m^2^, OX 100 mg/m^2^, and BEV 7.5 mg/m^2^), suggesting that further dose finding investigations are needed [15]. Accordingly, we planned the phase II QUATTRO-II study, which includes both dose finding to investigate the RDs (Step 1) and randomization to evaluate the efficacy and safety of CAPOXIRI+BEV versus FOLFOXIRI+BEV as a first-line treatment for mCRC (Step 2).

## Methods/design

### Study design and treatment

**Fig. 1:**
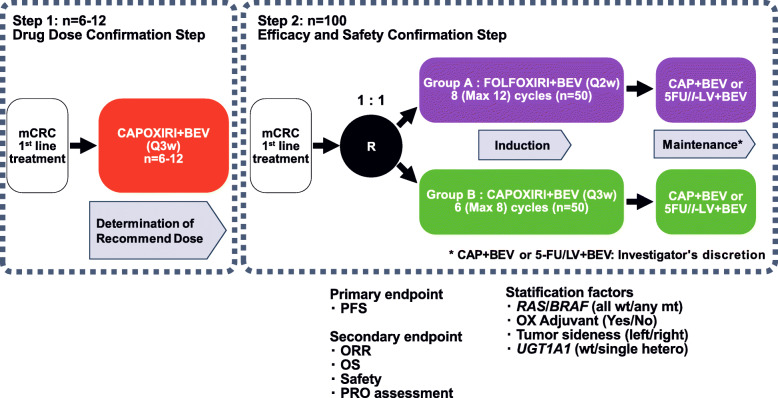
Graphical representation of the QUATTRO-II study design. mCRC, metastatic colorectal cancer; CAP, capecitabine; BEV, bevacizumab; OX, oxaliplatin; IRI, irinotecan; 5-FU/LV, fluorouracil and folinate; PFS, progression-free survival; ORR, overall response rate; OS, overall survival

The study design of QUATTRO-II is shown in Fig. 1. In Step 1, the RDs of OX and IRI will be investigated based on the doses previously specified in the phase III AXEPT study (IRI 200 mg/m^2^ and CAP 1600 mg/m^2^/day) during the first cycle. In Step 2, patients will be randomly assigned to FOLFOXIRI+BEV or RDs of CAPOXIRI+BEV.

Key eligibility criteria include patients aged over 20 years with unresectable colorectal adenocarcinoma with measurable lesions, Eastern Cooperative Oncology Group performance status (PS) of 0 or 1 (in patients aged ≥71 years, only PS 0 will be included), *RAS*/*BRAF* status diagnosed as either wild-type or mutant, wild-type (*UGT1A1* *1/*1), or single heterozygous type (*1/*6 or *1/*28) of *UGT1A1* polymorphism, adequate organ function, and no history of prior chemotherapy (complete inclusion and exclusion criteria are shown in Table 1).

**Table 1:** Patient inclusion and exclusion criteria

Inclusion criteria	
1. Personal written informed consent is obtained after the study has been fully explained	
2. Histologically confirmed colon or rectal adenocarcinoma (excluding appendix cancer and anal canal cancer)	
3. Clinically unresectable tumor	
4. ≥ 20 years of age at enrollment	
5. The ECOG performance status (PS) score of 0 or 1 (≥ 71 years of age: PS score of 0)	
6. Measurable lesion in accordance with RECIST ver. 1.1 criteria on contrast-enhanced chest, abdominal, or pelvic (trunk) CT (required within 28 days of enrollment)	
7. No previous chemotherapy for colon or rectal cancer (patients with confirmed relapse ≥24 weeks after completing post-operative adjuvant chemotherapy can be enrolled)	
8. *RAS*/*BRAF* mutation analysis at enrollment identifies *RAS*/*BRAF* status as either the wild-type or mutant type	
9. Vital organ functions meet the following criteria within 14 days before enrollment. If multiple test results are available in that period, the results closest to enrollment will be used. No blood transfusions or hematopoietic factor administration will be permitted within 2 weeks before the date on which measurements are taken. a. Neutrophil count: ≥ 1500/mm^3^ b. Platelet count: ≥ 10.0 × 10^4^/mm^3^ c. Hemoglobin concentration: ≥ 9.0 g/dL d. Total bilirubin: ≤ 1.5-fold the upper limit of normal (ULN) e. AST, ALT, ALP: ≤ 2.5-fold the ULN (≤ 5-fold the ULN for liver metastases) f. Serum creatinine: ≤ 1.5-fold the ULN, or creatinine clearance: ≥ 30 mL/min g. Urine protein: ≤ 2+ (if ≥3+, urine protein/creatinine ratio: < 2.0)	
10. *UGT1A1* polymorphism is wild-type or single heterozygous type	
Exclusion criteria	
1. Previous radiation therapy in which ≥20% bone marrow was exposed to the radiation field	
2. Untreated brain metastases, spinal cord compression, or primary brain tumor	
3. History of central nervous system disease (excluding asymptomatic lacunar infarction)	
4. Continuous systemic corticosteroid treatment is required	
5. Oral or parenteral (such as low molecular weight heparin) anticoagulant dose is not consistently (≥ 14 days) controlled (oral anticoagulants: conditions at high risk for bleeding, such as PT-INR ≥ 3, clinically significant active bleeding [within 14 days of enrollment])	
6. Arterial thrombosis or arterial thromboembolism such as myocardial infarction, transient ischemic attack, or cerebrovascular attack in the last year prior to enrollment	
7. Previous treatment with an investigational drug within 28 days before enrollment, or participation in a study of an unapproved drug	
8. Any of the following comorbidities: a. Uncontrolled hypertension b. Uncontrolled diabetes mellitus c. Uncontrolled diarrhea d. Peripheral sensory neuropathy (≥ Grade 1) e. Active peptic ulcer f. Unhealed wound (except for suturing associated with implanted port placement) g. Evidence of cardiovascular disease, cerebrovascular disorder (within 24 weeks), myocardial infarction (within 24 weeks), unstable angina pectoris, New York Heart Association classification ≥ Grade 2 congestive heart failure, serious arrhythmias requiring drug therapy h. Uncontrolled venous thromboembolism (unless clinically stable, asymptomatic, or appropriately treated with an anticoagulant) i. Systemic treatment required for, or evidence of, infections j. Other clinically significant diseases (such as interstitial pneumonia or renal impairment)	
9. Major surgical procedure within 28 days before study treatment initiation	
10. Physical defects of the upper gastrointestinal tract; malabsorption syndrome or difficulty taking oral medication	
11. Pregnant, breastfeeding, positive pregnancy test (women who have menstruated in the last year will be tested), or patients who are unwilling to use contraception during the study	
12. Active hepatitis B or C, or evidence of HIV infection	
13. Previous chemotherapy for other malignancies (excluding hormone therapy for breast cancer).	
14. Other active malignancies (excluding malignancies that are expected to be completely cured, such as intramucosal carcinoma and carcinoma in situ)	
15. Diseases such as intestinal paralysis, intestinal obstruction, or gastrointestinal perforation within 1 year prior to enrollment	
16. Pleural effusion, ascites, or pericardial effusion requiring drainage	
17. History of hypersensitivity to fluorouracil, levofolinate, oxaliplatin, irinotecan, bevacizumab, and their excipients or Chinese hamster ovary cell proteins	
18. History of adverse reactions to fluoropyrimidine drugs indicative of dihydropyrimidine dehydrogenase (DPD) deficiency	
19. Endoluminal stenting	
20. Otherwise unsuitable for the study in the opinion of the investigators	

This study is being conducted in accordance with Clinical Trials Act (Act No. 16 of April 14, 2017) in Japan, as well as with the ethical guidelines for medical and health research involving human subjects. All patients are required to sign written informed consent. We registered this study in Clinicaltrials.gov (NCT04097444) and Japan Registry of Clinical Trials (jRCTs041190072).

### Step 1

**Fig. 2:**
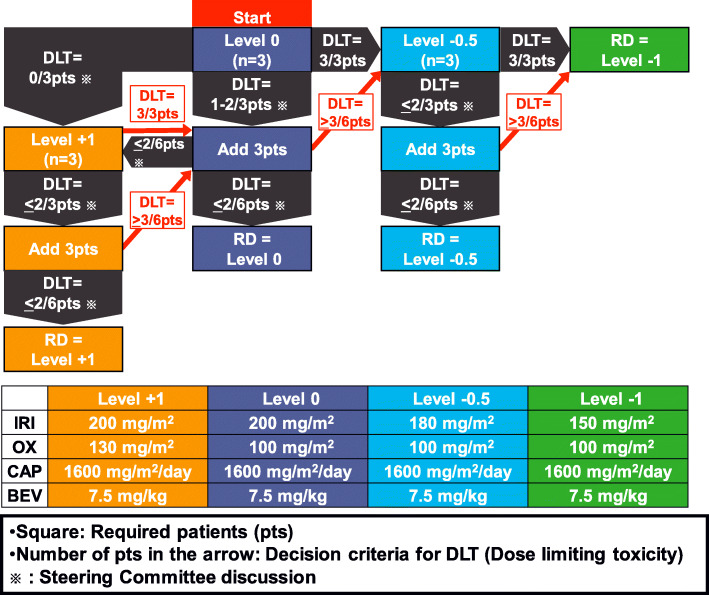
Drug dose confirmation step (Step 1). A dose confirmation part was established as Step 1 based on the doses in the AXEPT Study (CAP: 1600 mg/m^2^, IRI: 200 mg/m^2^). Steering Committee (SC) will assess dose limiting toxicity (DLT) for each dose level of OX and IRI in Cycle 1 (before the start of Cycle 2) to determine the recommended doses (RDs). After RD review by the Efficacy and Safety Assessment Committee, SC will report upon approval by the Certified Review Board. CAP, capecitabine; BEV, bevacizumab; OX, oxaliplatin; IRI, irinotecan

In Step 1, the RDs of CAPOXIRI will be determined in nine core hospitals. The dose schedule of CAPOXIRI+BEV is as follows; a 30–90-min infusion of BEV 7.5 mg/kg, 1-h infusion of IRI, 2-h infusion of OX, and 1–14 days of CAP 1600 mg/m^2^/day every 3 weeks. Four levels of CAPOXIRI doses (Level + 1 IRI 200 mg/m^2^, OX 130 mg/m^2^; Level 0 IRI 200 mg/m^2^, OX 100 mg/m^2^; Level − 0.5 IRI 180 mg/m^2^, OX 100 mg/m^2^; and Level − 1 IRI 150 mg/m^2^, OX 100 mg/m^2^) will be investigated in dose escalation or de-escalation analysis by including every three patients with reference to the decision process shown in Fig. 2. DLTs are defined as follows: (1) grade 4 neutropenia over 8 days, (2) febrile neutropenia, (3) grade 4 thrombocytopenia or grade 3 thrombocytopenia requiring platelet transfusion, and (4) ≥ grade 3 digestive symptoms that do not improve after ≥5 days despite optimal treatment. In each step, the steering committee (SC) will determine whether dose escalation or de-escalation should be performed and finally decide the RD of CAPOXIRI+BEV. Briefly, initially three patients will be treated with Level 0. In the case that DLTs are reported in 0/3 patients at Level 0, the three patients will be enrolled in Level + 1. When DLTs are reported in ≤2 of the three patients at Level + 1, three additional patients will be added at Level + 1. If DLTs are reported in ≤2 of six patients, Level + 1 will be determined to be the RD. If, however, DLTs are reported in 1–2 of the three patients at Level 0, or in 3/3 or ≥ 3/6 at Level + 1, three additional patients will be added to Level 0. These processes will be repeated from Level 0 to Level − 0.5, and from Level − 0.5 to Level − 1 in our 3 + 3 design (Fig. 2). After DLT assessment, induction with CAPOXIRI+BEV will be continued for up to 6 cycles (maximum of 8 cycles), followed by maintenance CAP+BEV or 5-FU/*l*-LV + BEV at the investigator’s discretion. After the review process of the Efficacy and Safety Assessment Committee, the study will proceed to Step 2.

### Step 2

In Step 2, patients will be randomly assigned to the FOLFOXIRI+BEV (Arm A) or recommended doses of CAPOXIRI+BEV (Arm B) using a minimization method. Participating institutions will be expanded to 25 hospitals. The stratification factors for randomization are as follows: *RAS*/*BRAF* (all wild-type/any mutant), OX adjuvant (yes/no), tumor sidedness (left/right), and *UGT1A1* (wild-type/single hetero).

**Fig. 3:**
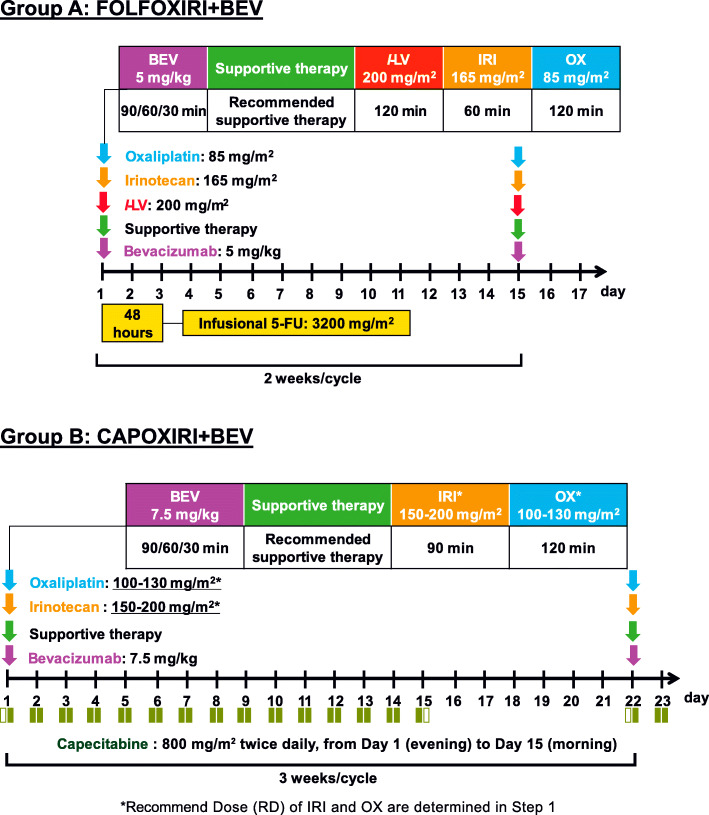
Induction therapy of FOLFIRI+BEV and CAPOXIRI+BEV (Step 2). FOLFOXIRI+BEV (bi-weekly) will be repeated 8 cycles (max: 12 cycles). CAPOXIRI+BEV (tri-weekly) will be repeated 6 cycles (max: 8 cycles), in which OX and IRI dose levels are determined by Step 1. The use of supportive therapy during protocol induction therapy is strongly recommended. CAP, capecitabine; BEV, bevacizumab; OX, oxaliplatin; IRI, irinotecan; 5-FU/LV, fluorouracil and folinate

**Fig. 4:**
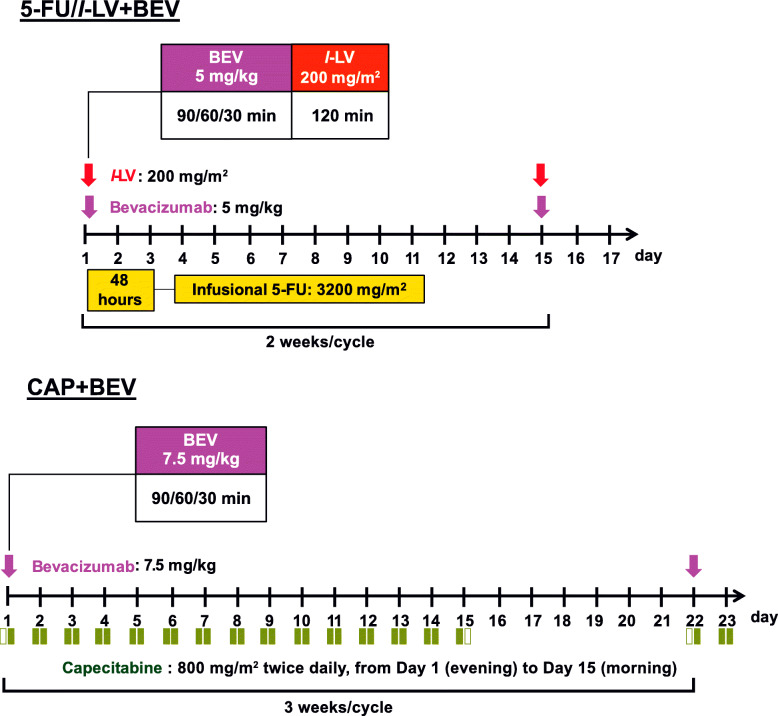
Maintenance therapy of 5-FU/LV + BEV and CAP+BEV (Step 2). 5-FU/LV + BEV or CAP+BEV will be selected by investigators during the protocol maintenance therapy. After selecting the regimen, no change of drugs is permitted. The protocol treatment will be discontinued when the primary disease progresses or when the protocol treatment cannot be continued because of adverse events or at the patients’ request. CAP, capecitabine; BEV, bevacizumab; OX, oxaliplatin; IRI, irinotecan; 5-FU/LV, fluorouracil and folinate

The treatment of Arm A involves induction FOLFOXIRI+BEV and maintenance CAP+BEV or 5-FU/*l*-LV + BEV (Figs. 3 and 4). The dose schedule of FOLFOXIRI+BEV is as follows: 30–90-min infusion of BEV 5 mg/kg, 1-h infusion of IRI 165 mg/m^2^, 2-h infusion of OX 85 mg/m^2^, *l*-LV 200 mg/m^2^, and 48-h continuous infusion of 5-FU 3200 mg/m^2^ every 2 weeks, which is the same as that of the TRIBE phase III and QUATTRO phase II studies [13]. Supportive therapy includes a 30-min infusion of palonosetron 0.75 mg on day 1, dexamethasone 9.9 mg on day 1 followed by 8 mg on days 2–4, and oral aprepitant 125 mg on day 1 followed by 80 mg on days 2–3 (or a 30-min infusion of fosaprepitant meglumine 150 mg on day 1). Induction therapy of FOLFOXIRI+BEV will be repeated for up to 8 cycles (maximum of 12 cycles), followed by maintenance 5-FU/*l*-LV + BEV (BEV 5 mg/kg, *l*-LV 200 mg/m^2^, and 48-h continuous infusion of 5-FU 3200 mg/m^2^ every 2 weeks) or CAP+BEV (BEV 7.5 mg/kg, CAP 1600 mg/m^2^/day every 3 weeks) until disease progression or unacceptable toxicities. A change from 5-FU/*l*-LV + BEV to CAP+BEV and vice versa in the maintenance period will not be allowed.

The treatment of Arm B comprises the induction CAPOXIRI+BEV and maintenance CAP+BEV or 5-FU/*l*-LV + BEV (Figs. 3 and 4) with use of the above supportive therapy. The RDs of CAPOXIRI+BEV will be repeated for up to 6 cycles (maximum of 8 cycles), and the following maintenance 5-FU/*l*-LV + BEV or CAP+BEV will be continued as in Arm A.

In both arms, surgical resections will be strongly recommended when optimal tumor shrinkages are observed every 8 ± 2 weeks of evaluation. Protocol treatments will be terminated when surgical treatments are performed.

### Endpoints and assessments

The primary endpoint of this study is PFS in Step 2. The secondary endpoints are ORR, OS, incidence of adverse events (AEs), and PRO. The response will be determined by computed tomography (CT) scanning based on Response Evaluation Criteria in Solid Tumors version 1.1. CT evaluations will be performed once every 8 weeks (±2 weeks) for up to 72 weeks, and then once every 12 weeks (±2 weeks). We define PFS as the period from registration to progression or death from any cause and will censor this time on which the last day the patient is alive without progression. AEs will be assessed according to the Common Terminology Criteria for Adverse Events version 5.0. PRO assessment for PSN will be performed using the FACT/GOG-Ntx4 questionnaire.

### Target sample size and statistical analyses

The sample size of the QUATTRO-II study will be determined based on the 75.2% PFS rate at 10 months in the QUATTRO study and 50% PFS rate at 12 months in the TRIBE study [10, 13]. The similarity of PFS between Arms A and B will be evaluated by observing whether the point estimate of the HR for PFS falls between 0.80 and 1.25. Ensuring a 70% probability that the observed HR will be “0.8 < HR < 1.25” under the assumption of a true HR of 1.0 and piecewise exponential distribution characterized by a 75% PFS rate at 10 months and 50% PFS rate at 12 months, 100 patients (50 patients in each arm) will be required for the 3-year study period. PFS and OS will be estimated by Kaplan–Meier analysis. The treatment response and other secondary endpoints among subgroups will be summarized using appropriate analytical methods.

## Discussion

FOLFOXIRI+BEV is the most effective regimen for mCRC; however, the management of its AEs including hematologic toxicities remains difficult [10]. Our QUATTRO study of FOLFOXIRI+BEV also reported a high incidence rate of grade 3 or 4 severe neutropenia of 72.5%, suggesting a high degree of hematologic toxicities in both Caucasian and Asian populations [10, 13]. Thus, novel regimens are needed to address this problem. A reduced dose of CAP in dual and triple combination regimens has attracted attention for managing AEs while maintaining efficacy. The phase II AIO0604 Study (IRI 200 mg/m^2^ and capecitabine 1600 mg/m^2^/day) showed that modified CAPIRI+BEV was as effective as CAPEOX+BEV and resulted in fewer AEs [16]. The AXEPT trial by Xu et al. (IRI 200 mg/m^2^ and capecitabine 1600 mg/m^2^/day) revealed a significantly lower incidence of grade 3 or 4 hematological AEs with modified CAPIRI+BEV than with FOLFIRI+BEV without impairing efficacy. These results suggest that CAPOXIRI+BEV with a reduced dose of CAP and RDs of OX and IRI can be equal to, or better than, FOLFOXIRI+BEV in terms of safety, feasibility, and efficacy. However, one concern associated with CAPOXIRI+BEV is the higher rate of gastrointestinal toxicities including diarrhea. According to a Japanese phase I study reported by Sato et al., CAPOXIRI+BEV, with a lower dose of irinotecan (150 mg/m^2^) than our regimen (200 mg/m^2^), showed 8% (1/12) grade 3 diarrhea. Although slightly higher rates of diarrhea is estimated with our regimen, it is expected to be manageable with appropriate supportive care [15].

As PSN is a clinically significant AE associated with continuous OX administration, assessment of PSN is one of the secondary endpoints in this study. Measures such as the administration of Ca/Mg, carbamazepine, duloxetine, and pregabalin have been considered for managing PSN and improving patients’ quality of life (QOL); however, no study has reported adequate evidence for this effect [17–20]. The ACHIEVE trial was conducted in Japan to compare 6 months of either CAPEOX or FOLFOX versus 3 months of the same regimens as adjuvant chemotherapy, which revealed a significantly lower percentage of PSN lasting 3 years in CAPEOX than in the FOLFOX group (7.9% versus 15.7% in 3-month arms; *p* = 0.04 and 21.0% versus 34.1% in 6-month arms; *p* = 0.02) [21]. Therefore, the tri-weekly dosing schedule including CAPOXIRI may contribute to the mitigation of OX-induced PSN.

FOLFOXIRI+BEV has other disadvantages in terms of continuous intravenous infusion, port placement, and visit frequency. Patients treated with bi-weekly regimens including FOLFOXIRI require more hospital visits for drug administration and spend more time in the hospital than those treated with tri-weekly regimens including CAPOXIRI. In addition, cost-minimization analysis showed that the total cost of chemotherapy and total disease management cost per patient in CAPEOX were significantly lower than those in FOLFOX [22]. CAPOXIRI+BEV is a tri-weekly regimen without the necessities of port placement and infusion pumps on an outpatient basis; this is expected to be an easier and safer treatment, with better QOL and lower medical costs. The exploratory confirmation of the safety and efficacy of CAPOXIRI+BEV versus FOLFOXIRI+BEV in this study will provide evidence and a new treatment option for the first-line treatment of mCRC.

## Data Availability

Not applicable.

## Abbreviations

5-FU: 5-Fluorouracil
AE: Adverse event
BEV: Bevacizumab
Ca: Calcium
CAP: Capecitabine
CAPIRI: Capecitabine+irinotecan
CAPEOX: Capecitabine+oxaliplatin
CAPOXIRI: Capecitabine+oxaliplatin+irinotecan
CT: Computed tomography
DLT: Dose limiting toxicity
ECOG: Eastern Cooperative Oncology Group
FACT: Functional assessment of cancer therapy
FOLFIRI: 5-Fluorouracil+levofolinate calcium+irinotecan
FOLFOX: 5-Fluorouracil+levofolinate calcium+oxaliplatin
FOLFOXIRI: 5-Fluorouracil+levofolinate calcium+oxaliplatin+irinotecan
HIV: Human immunodeficiency virus
HR: Hazard ratio
IRI: Irinotecan
l-LV: Levofolinate calcium
mCRC: Metastatic colorectal cancer
Mg: Magnesium
NCCN: National Comprehensive Cancer Network
NYHA: New York Heart Association
ORR: Overall response rate
OS: Overall survival
OX: Oxaliplatin
PD: Progressive disease
PFS: Progression-free survival
PRO: Patient reported outcome
PS: Performance status
PSN: Peripheral sensory neuropathy
QOL: Quality of life
RD: Recommended dose
UGT: Uridine diphosphate glucuronosyltransferase
VEGF: Vascular endothelial growth factor
